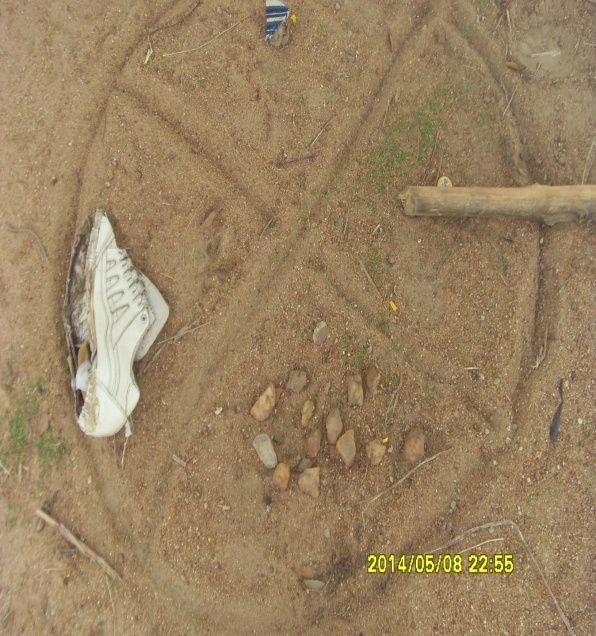# Listening to the Voices of the People: Community’s Assessment of Disaster Responder Agency Performance During Disaster Situations in Rural Northern Ghana

**DOI:** 10.1371/currents.dis.4226abe816b2746df13d16ea307b5846

**Published:** 2017-09-20

**Authors:** Stephen Apanga, Gregory Titi Addebah, Dennis Chirawurah

**Affiliations:** Department of Community Health and Family Medicine School of Medicine and Health Sciences, University for Development Studies Tamale Ghana; School of Medicine and Allied Health Sciences, University for Development studies, Tamale, Ghana; ResilientAfrica NetworkUniversity for Development Studies

## Abstract

**Introduction:**

In Northern Ghana, a combination of torrential rains coupled with the spilling of the Bagre dam in neighboring Burkina Faso in the past few years has resulted in perennial flooding of communities. This has often led to the National Disaster Management Organization (NADMAO) the main disaster responder agency in Ghana, being called upon to act. However affected communities have never had the opportunity to evaluate the activities of the agency. The aim of this study is therefore to assess the performance of the main responder agency by affected community members to improve on future disaster management.

**Methods:**

A mixed qualitative design employing a modified form of the community score card methodology and focus group discussions was conducted in the 4 most affected communities during the last floods of 2012 in the Kasena-Nankana West district of the Upper East Region of Northern Ghana. Community members comprising of chiefs, elders, assembly members, women groups, physically challenged persons, farmers, traders and youth groups formed a group in each of the four communities. Generation and scoring of evaluative indicators was subsequently performed by each group through the facilitation of trained research assistants. Four Focus group discussions (FGDs) were also conducted with the group members in each community to get an in-depth understanding of how the responder agency performed in handling disasters.

**Results:**

A total of four community score cards and four focus group discussions were conducted involving 48 community representatives. All four communities identified NADMO as the main responder agency during the last disaster. Indicators such as education/awareness, selection process of beneficiaries, networking/collaboration, timing, quantity of relief items, appropriateness, mode of distribution of relief items, investigation and overall performance of NADMO were generated and scored. The timing of response, quantity and appropriateness of relief items were evaluated as being poor whereas the overall performance of the responder agency was above average.

**Conclusion:**

NADMO was identified as the main responder agency during the last disasters with community members identifying education/awareness, selection process of beneficiaries, networking/collaboration, timing of response, quantity of relief items, appropriateness of relief items, mode of distribution of relief items, investigation and overall performance as the main evaluative indicators. The overall performance of NADMO was rated to be satisfactory.

**Key words:**

Kasena-Nankana West district, NADMO, community score card, Rural Northern Ghana

## INTRODUCTION

Over the past several decades, both naturally occurring and man-made disasters have increased in frequency and number worldwide with Sub-Saharan Africa considered to be the most vulnerable to climate variability in particular.^1^^,^ 2 The growing frequency and severity of extreme events such as droughts and floods along with shifting rainfall patterns, threaten to overwhelm the natural resilience of African communities risking livelihoods and food security. Widespread poverty, fragile ecosystems, weak institutions, and uncoordinated disaster response among responding agencies exacerbate Africa’s vulnerability to climate change.^3^^,^ 4

In Northern Ghana, a combination of torrential rains coupled with the spilling of the Bagre dam in neighboring Burkina Faso in 2007, 2010 and 2012 spelled doom for the area. According to the UN Office for the Coordination of Humanitarian Affairs, the 2007 floods alone killed 22 people, destroyed or completely damaged 11,239 homes, washed away 12,220 hectares of farmland and affected 275,000 people.^5^

As the reported number of people affected by disasters has risen over the past decades, so has the expectations placed on responding agencies by donors, the public and affected populations also increased.^6^ In contrast to financial accountability mechanisms which are now comparatively well developed within donor and implementing agencies, the mechanisms for ensuring accountability to the population being served are poorly developed. Apart from the fact that there is the need to improve accountability of relief interventions through the development of appropriate criteria and methods to measure their successes, there is perhaps more importantly, the need for the creation of a management culture which exacts the high standards of accountability from donor and implementing agencies to recipient communities.^7^

The Kasena Nankana west district was one of the hardest hit in all the previous disastrous events resulting in widespread humanitarian effort by government; non-governmental organizations (NGOs) such as the International Federation of the Red Cross and the Inter-NGO Consortium; and individuals. All the humanitarian aid was channeled through the National Disaster Management Organization (NADMO) the main disaster responder agency in Ghana established by act 517 of 1996 to manage disasters and similar emergencies in the country.^8^ However there have been a number of allegations such as over concentration of relief items to areas that were not hardest hit, unfair distribution of relief items and poor coordination of the disasters among others labeled against NADMO by affected community members.

There is currently scarcity of knowledge concerning the performance of the main disaster responder agency by community members in Northern Ghana especially after the disastrous floods of 2007, 2010 and 2012. We therefore conducted this study to assess the performance of the main responder agency at the grass root level in Northern Ghana and to set the platform for an interactive feedback between responder agency and community members for future disaster management.

## METHODS

**Study area and Design**

This study was conducted from May to June 2014 in the four most affected communities (Navio, Kajelo Nyangenia and Nakong) of the last floods of 2012 in the Kasena-Nankana West district of the Upper East Region of Northern Ghana. The Kassena-Nankana West District is one of the thirteen districts in the Upper East Region of Ghana. Its population according to the 2010 Population and Housing Census is 70,667 representing 6.8 percent of the region’s total population. Males constitute 50.8 percent and females represent 49.2 percent. Seventy nine percent of the population is rural. The population is engaged in various economic and social activities including; subsistence farming, business/trade, rearing domestic animals among others.^9 ^

The map of the Kassena-Nankana West District showing the study communities is shown in Figure 1.

**Map of Kassena-Nankana West District showing study communities:**
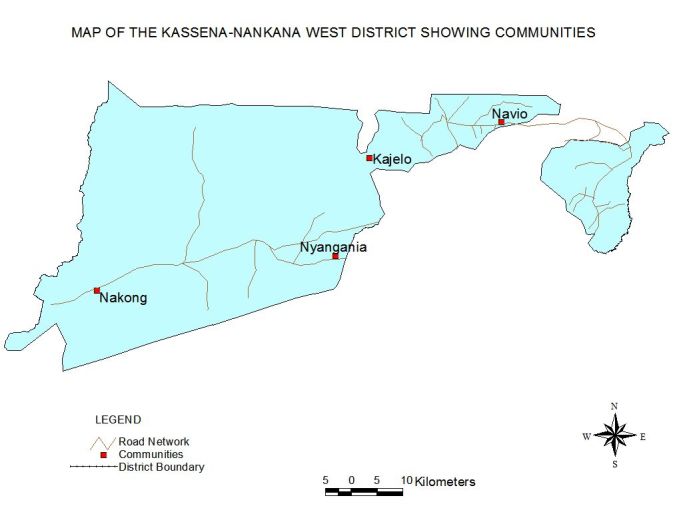

Due to the growing interest and importance in the use of qualitative, especially participatory techniques in evaluating relief programs^7^, a qualitative design employing a modified form of the community score card methodology and focus group discussions were used in this study.

**The Community Score Card methodology**

The community score card (CSC) process is a community-based monitoring tool that is a hybrid of the techniques of social audits and citizen report cards. The CSC is an instrument to exact social and public accountability and responsiveness from service providers. By linking service providers to the community, citizens are empowered to provide immediate feedback to service providers. The CSC process uses the “community” as its unit of analysis, and is focused on monitoring at the local/facility level. It can therefore facilitate the monitoring and performance evaluation of services, projects and even government administrative units by the community themselves. Since it is a grassroots process, it is also more likely to be of use in a rural setting. CSC process involves basically four components: input tracking scorecard, community generated performance scorecard, self-evaluation scorecard by service providers and an interface meeting between users and providers to provide respective feedback and generate a mutually agreed reform agenda.^10^^,^ 11^,^ 12

For the purpose of this study, we employed only the component of community generated performance scorecard as this involves the generation of evaluative indicators by community members themselves.

**Selection of study participants**

At the community level, members comprising of chiefs, elders, assembly members, women groups, physically challenged persons, farmers, traders and youth groups formed a group in each of the four communities. This target population was those actually chosen by community members to represent them in the communities’ dealings with the responder agency during the disasters. Each group was considered to be heterogeneous since the members were from different backgrounds, ages and genders. All participants were however aged 18 years and above.

**Data collection process and analysis**

Two experienced research assistants were trained on the community score card approach, the study protocol and procedures to assist the investigators in data collection.

In all, four community visits where made to each community by the research team. The first visit was to explain the purpose of the study to the chiefs, elders and opinion leaders of the communities. During the second community visit, a community meeting was held where the purpose of the study and scorecard methodology (with much emphasis on indicator generation) was explained to community members after which each community chose its own group members. On the third visit, the field research team facilitated an indicator-generation process where groups generated and defined a set of indicators. The definitions of indicators were given entirely by group members themselves after which group representatives from each group met to consolidate and harmonize these definitions under the guidance of the research team. In the final community visit, group members met to score each indicator where a circle was drawn on the ground and divided into different sections by one of the participants in each group. Items like sticks, sandals and stones were used to denote an indicator in each of the divided sections of the circle. Participants cast a small stone each in the divisions of the circle (figure 2) to represent their individual grading/score of an indicator as arrived at by consensus. The groups further settled on a collective scoring measure or rating of 1-100 to score some indicators. These scoring processes in the form of a scoring matrix were then summarized into a score card and presented in the form of tables.

Focus group discussions (FGDs) were also conducted with the group members in each community during the third community visit to get an in-depth understanding of how the responder agency performed in handling disasters. FGDs were recorded with an audio tape and later transcribed verbatim from the local language (kassem) into English in a text form for analysis. The investigators read through all the transcripts exhaustively to check for inconsistencies after which coding and categorization was done manually using the conventional analysis approach as outlined by Hsiu-Fang and Sarah.^13^ Categories were then grouped under thematic areas which corresponded with community generated indicators by the community score card approach for the write-up.

**Ethical considerations**

Ethical approval was given by the Navrongo Health Research Centre Institutional Review Board (ID: NHRCIRB 183) and Kasena-Nankana West district assembly and district NADMO office. Permission to carry out this survey at the community level was verbally sought from the chiefs and elders of the respective communities first, after which a verbal informed consent was also gotten from individual members of the participating groups. The consent procedure was reviewed by and approved by the ethics committee.

## RESULTS

In total four community score cards and four focus group discussions were conducted involving 48 community representatives. Males accounted for 56% (27) of the population with the remaining 44% (21) being females. The average age of participants was 52 years. An average of 12 members formed a group in each of the communities.

Findings of both the community score card and focus group discussions are presented together.

**Community Score Cards**

All four communities identified NADMO as the main responder agency during the last and previous disasters. Community generated indicators included: Education/awareness, Selection process of beneficiaries, Networking/Collaboration, Timing of response, Quantity of relief items, Appropriateness of relief items, Mode of distribution of relief items, Investigation and Overall performance of NADMO.

The consolidated and harmonized definitions of the indicators is presented in table 1 whereas the summarized score card (individual and consolidated scoring) for each of the communities are presented in tables 2 to 5.

**Table 1. DEFINITION OF INDICATORS:** 

INDICATOR	DEFINITION
Education/awareness	The extent to which people were educated/made aware on flooding, storms etc in the community using durbars, radio or other means; sensitizing communities and not waiting for disasters before you intervene
Selection process of beneficiaries	Degree of satisfaction on who will benefit from relief items and the selection criteria of beneficiaries
Investigation	Assessing degree of destruction and its accompanying effects on affected people for deciding on who gets what and by how much
Timing	The time between the writing of names of affected persons and when the relief is brought to them
Quantity of relief items	The amount of relief given in relation to what the victim actually needed/required
Appropriateness	Whether relief given is what the victim needed/ lost due to disaster
Mode of distribution	Mode of distribution of relief breeds conflict among those who get and those who do not
Networking	Assessing who/which entities collaborated in assessing, deciding and distributed relief items to affected persons
Overall performance	NADMO overall performance graded

**TABLE 2. SCORE CARD FOR NAVIO COMMUNITY:** 

INDICATOR	UNIT OF MEASUREMENT	INDIVIDUAL SCORES	CONSOLIDATED SCORES (1-100)
Education/awareness	Media/radio	11	NOT APPLICABLE
	Community durbars	0	
	No education	1	
Selection process of beneficiaries	Good	0	20
	Poor	0	
	Very poor	10	
	No investigation	2	
Networking/Coordination	Involves a committee	0	NOT APPLICABLE
	External distributors	2	
	NADMO officials	8	
	Party leaders	2	
Timing	Timely	0	20
	Late/average	2	
	Very late	10	
	Never came	0	
Quantity of relief items	Adequate	2	30
	Inadequate	2	
	Woefully inadequate	4	
	Not at all/Absent	4	
Appropriateness	Appropriate	0	50
	Average	2	
	Somehow	9	
	Not at all	1	
Overall performance	Good	7	60
	Average	2	
	Poor	3	

**TABLE 3. SCORECARD FOR NAKONG COMMUNITY:** 

INDICATOR	UNIT OF MEASUREMENT	INDIVIDUAL SCORES	CONSOLIDATED SCORES (0-100)
Education/awareness	Media/radio	0	NOT APPLICABLE
	Community durbars	0	
	No education	12	
Selection process of beneficiaries	Excellent	12	100
	Good	0	
	Poor	0	
Mode of distribution	Satisfactory	0	10
	Not satisfactory	12	
Timing	Timely	0	20
	Late/average	0	
	Very late	7	
	Never came	5	
Quantity of relief items	Adequate	0	10
	Inadequate	0	
	Woefully inadequate	12	
	Not at all/Absent	0	
Appropriateness	Appropriate	0	10
	Average/inappropriate	0	
	Woefully inappropriate	12	
Overall performance	Satisfactory	6	50
	Somehow satisfactory	6	

**TABLE 4. SCORE CARD OF NYANGANIA COMMUNITY:** Responses where based on earlier encounters with NADMO and not the recent 2012 floods

INDICATOR	UNIT OF MEASUREMENT	INDIVIDUAL SCORES	CONSOLIDATED SCORES (0-100)
Education/awareness	Media/radio	1	NOT APPLICABLE
	Community durbars	0	
	No education	11	
Timing	Timely/appropriate	0	1
	Inappropriate	0	
	Somehow	0	
	Not at all	12	
Quantity of relief items	Adequate	0	1
	Inadequate	0	
	Woefully inadequate	0	
	Not at all/Absent	12	
Overall performance	Good	0	50
	Average	6	
	Poor	6	

**TABLE 5. SCORE CARD OF KAJELO COMMUNITY:** 

INDICATOR	UNIT OF MEASUREMENT	INDIVIDUAL SCORES	CONSOLIDATED SCORES (0-100)
Education/awareness	Regular education	1	60
	No education	3	
	Somehow	8	
Selection process of beneficiaries	Very good	0	40
	Good	5	
	Poor/selective	7	
Investigation	Proper	0	30
	Poor	6	
	Not at all	6	
Timing	Timely	0	20
	Late/average	1	
	Very late	11	
Quantity of relief items	Adequate	0	30
	Inadequate	12	
	Woefully inadequate	0	
Appropriateness	Appropriate	0	30
	Inappropriate	12	
	Somehow	0	
Overall performance	Satisfactory	4	40
	Unsatisfactory	8	

**Focus Group Discussions**

***Education/awareness***

Two of the communities (Nyangania and Nakong) said they did not receive enough education and sensitization on the flooding situation resulting in community members being hit hard by the floods. This they said also affected their resilience level adversely.

“The local FM station did a good job by constantly announcing and reminding us of when they were going to open the dam from neighboring Burkina Faso. This helped members of the community to prepare adequately and so we were able to adapt to the floods quite well with the help of NADMO” (Group member of Navio community-FGD)

“Our community did not receive information or education from any organization. So when the flood came we were not prepared at all and this led to loss of lives and properties of community members” (Group member of Nakong community-FGD)

***Section process of beneficiaries***

Members of three communities (Navio, Nakong and Kajelo) identified this as an indicator with only one of these communities (Nakong) rating this indicator high.

“In fact we were really very satisfied with how NADMO selected those who were to benefit from the relief items they brought to us” (Group member of Nakong community-FGD)

“The way NADMO selected those who were to benefit from the relief items was poor. For example some of the affected people who wrote done their names did not receive any item whiles some people who didn’t write their names at all were given relief items” (Group member of Kajelo community-FGD)

***Networking***

This indicator was identified by only one community with members of the group identifying the existence of networking and collaboration between NADMO officials and other stakeholders in the management of the last disasters.

“NADMO officials, leaders of some political parties and other organizations who donated relief items to us worked together in deciding those who got those items” (Group member of Navio community-FGD)

***Timing of response***

Whereas three of the communities (Navio, Nakong and Kajelo) got some response from the responder agency, the response rather arrived very late with the fourth community (Nyangania) not receiving any response at all during the last floods in 2012.

“Although we eventually got some items such as cups, mattresses, blankets and buckets from NADMO, these items arrived very late. We hope next time there is a disaster such as this, NADMO and any organization which has items to donate, will do that as quick as possible to help the community recover more quickly” (Group member of Kajelo community-FGD)

“For the last floods that we experienced here, NADMO visited us to write the names of those who were affected through the community elders. However they never came back again and so no single person received any relief item in this community” (Group member of Nyangania community-FGD)

***Quantity of relief items***

Majority of respondents from communities were the responder agency responded opined that the quantity of relief items were generally inadequate.

“The quantity of relief items we received was not enough at all. Imagine after writing down the names of so many affected people only 8 mattresses were distributed to the worst affected areas of Kajelo through the elders of the community” (Group member of Kajelo community-FGD)

Appropriateness of relief items

Those communities that got a response from NADMO rated the appropriateness of this response to be inadequate. All the 3 communities that got some help from NADMO said the relief items they received were exactly what they needed to cope and recover from the floods.

“Though officials of NADMO came to write our names and the items we actually needed, what they eventually brought to us was different and this did not help us that much especially on how we coped and recovered from the floods” (Group member of Navio community-FGD)

***Mode of distribution of relief items***

Only one (Nakong) community evaluated the responder agency using this as an indicator with group members stating that the mode of distributing relief items was unsatisfactory.

“The unfair manner with which the relief items were distributed to affected members of the community rather brought about some disagreements and conflicts amongst the beneficiary since some people thought they were most affected and therefore deserved more items. Some members of this community have also come to complain to me that the relief items were distributed along partisan lines and not based on merit” (Group member of Nakong community-FGD)

***Investigation***

Group members in only one (Kajelo) community identified community needs assessment by the responder agency as an evaluative indicator with 50% of the members saying that this was poorly done and the other 50% saying this was not carried out at all.

“Although NADMO came around to assess the extent of damage by the floods before writing the names of those to benefit from any relief item in my area, this was poorly done as some of the people who were seriously affected didn’t get the right quantity of items to help them deal with the situation” (Group member of Kajelo community-FGD)

“NADMO never did any investigation or assessment of the level of damage by the floods in my locality before writing down the names of those affected. And so some of those who were not seriously affected got the same items as those who were seriously hit by the floods” (Group member of Kajelo community-FGD)

***Overall performance***

All four communities evaluated NADMO using this as an indicator. Apart from one (Kajelo) community that rated the overall performance of NADMO to be unsatisfactory, the rest of the communities thought that NADMO performed creditably well in its handling of the recent disasters.

“I will say that overall, NADMO performed quite well when you look at how they handled the entire disaster situation” (Group member of Navio community-FGD)

“Even though NADMO did not respond to our needs this time round, judging by what they did during the previous floods I will say that their performance was satisfactory” (Group member of Nyangania community-FGD)

“I wasn’t happy with the performance of NADMO at all. They were not fair with the way they responded to this particular disaster. Those who really needed the items never got them and for those who eventually received their items got them very late. So how can you say that NADMO did well?” (Group member of Navio community-FGD)

## DISCUSSION

The methodology employed in this study can be said to be one of the new emerging participatory techniques used in evaluating relief programs and agencies especially against the background that the CSC is being used for the first time for this purpose.

The Hyogo World Conference on Disaster Reduction in 2005 stressed on the importance of information management and exchange which is meant to provide easy understanding of information on disaster risks and protection options, especially to citizens in high-risk areas, so as to encourage and enable people to take action to reduce risks and build resilience.^14^ However in this setting, half of the affected communities did not receive sufficient information and education on how to reduce their risk and were hit severely by floods which affected their resilience adversely. Disaster management at the community level involves the need for information flow in a coordinated fashion through both a multi-organizational and multi-level structure, requiring that responder agencies or organizations not only depend on their internal interactions, but on the interactions with other agencies as well.^15^ NADMO over the years has worked hand in hand with other agencies in achieving one of its objectives which is to set up monitoring and early warning systems to aid the identification of disasters in their formative stages, to disseminate timely information and warning, and hazard/disaster awareness creation.^8^ However during this disaster, this was not the case as information dissemination and awareness creation was generally found to be unsatisfactory by community members. It is important that the responder agency takes advantage of the privileges at its disposal especially during disaster situations to embark on massive community awareness creation and information dissemination on how communities can mitigate, adapt and cope with the shocks and stressors of disasters.

Selection of beneficiaries during and after disasters can be a very daunting task. In this particular disaster, majority of the community members labeled a number of allegations against the responder agency. Similarly in a qualitative study to evaluate post-flood disaster response strategies in southern Ghana, some male participants in a focus group discussion also accused the same disaster responder agency as being discriminatory and actually decided not to violently contest the seeming discrimination they felt so as to sustain the unity in their community.^16^

Post-disaster governmental manipulations of response processes has been identified to exacerbate existing inequalities within affected communities which has often tend to provoke angry protest and demonstration against the institutions in charge.^17^^,^ 18 In one of the communities in this study, members of a focus group discussion complained of how the responder agency was being manipulated by the ruling government leading to some disagreements and conflicts amongst community members due to the distribution of relief items along partisan lines.

In non-disaster situations, many of the agencies involved in disaster management operate independently of each other. At the community level in a disaster situation however, this is often not the case as complexity arises from a variety of elements, systems, processes and actors in a complex network of many interactions between these agencies at various levels necessary for achieving mutual adjustment and a collective goal.^15^^,^^19^^,^^20^^,^^21^ Although a number of agencies were involved in managing the floods in the Kasena Nankana west district of northern Ghana by assisting the main responder agency, majority of the affected communities did not see any tentative collaboration or networking between the various actors. Effective networking and collaboration at the community level in particular can serve as a basis of community members developing confidence in disaster management and also help reduce the number of allegations labeled against the responder agency.

It is a usual practice for NADMO to register victims of disasters before resources are mobilized and sent to the community.^8^ This process however tends to take a long time resulting in delays in supplying relief items as is common with most disaster management agencies.^22^^,^^23^ According to the United Nations, much of the lives loss during a disaster or hazard event occurs in the first 24-48 hours^24^ and therefore the delays on the part of NADMO in its response as a result of pre- registration of affected people can usually be catastrophic. During the 2012 flood disaster, all the communities which eventually got assistance from NADMO identified the timing of this assistance especially distribution of relief items to be late after most community members where already badly hit by the floods. This was also the situation in southern Ghana when NADMO delayed in distributing relief resources to registered flood victims in 2010.^16^

In one of the study communities, the need to conduct a needs assessment during and after disasters was identified as evaluative indicator as community members thought this was crucial especially concerning the distribution of relief items by the responder agency. This point has also been emphasized by Oaks in an Overview of the Damage Assessment Process: as he suggests that, after disasters, the damage assessment process is fundamental to relief and reconstruction as it triggers the beginning of formalized disaster relief and recovery aid.^25^ Similarly research has also shown that, damage assessment helps amongst other things to identify the immediate needs of disaster victims, whiles enabling first responders and emergency managers to recognize required materials and human resources.^26^

Majority of group members in the affected communities in the Kasena Nankana west district of northern Ghana rated the overall performance of the responder agency to be above average. Although some group members rated certain specific generated indicators low, they thought that in the whole, the responder agency performed creditably well. This finding only emphasis the fact that community members tend to assess performance when certain specific needs are met and not just an overall scoring scale of evaluative indicators as is usually found in quantitative studies. However contrary to the findings of our study, an earlier qualitative study involving the same responder agency found the overall performance of the agency to be poor after the catastrophic 2010 flooding situation in southern Ghana.^16^ Similarly in Ekiti State in Nigeria, the performance of the State Emergency Management agency which is the main disaster responder agency was found to be comparatively poor.^27 ^

The observed differences in the results across the four communities can be attributed to the fact the these communities are often affected differently by the perennial floods with the response from the responder agency being different depending on the level of preparedness by NADMO at the local level and availability of support from all stakeholders.

**Limitations**

Notwithstanding the strong qualitative methodological approach adapted by this study, the study had some limitations. We conducted the study in only four communities and about two years after the disaster occurred and therefore the accuracy and precision of the information provided could have been affected especially that some of the community leaders would have relocated, died or forgotten certain vital events that occurred.

Although the composition of the groups was heterogeneous, group members were only elders and representatives of the communities and therefore their opinions might not necessarily be the views of the ordinary members of the communities who were hit hardest by the floods.

## Conclusion

This study used a combined qualitative methodological approach of focus group discussions and the community score card in four communities in Northern Ghana to evaluate the performance of the main disaster responder agency. All four communities identified NADMO as the main responder agency during the last disasters with community members identifying education/awareness, selection process of beneficiaries, networking/collaboration, timing of response, quantity of relief items, appropriateness of relief items, mode of distribution of relief items, investigation and overall performance as the main evaluative indicators. Although the indicators were rated differently by each community; the timing of response, quantity and appropriateness of relief items were generally poor whereas the overall performance of the responder agency was generally satisfactory.

Since these communities are often prone and vulnerable to the perennial floods, we recommend that the main responder agency responds more timely and fast whiles engaging the other stake holders in disaster management to ensure that communities get enough of the most appropriate relief items. We also encourage that after each disaster, all the stake holders in disaster management great that platform were the disaster responder agency will be evaluated by the affected communities as a feedback to improve on future disaster management

## Competing Interests

The authors have declared that no competing interests exist.

## Financial Disclosure

This study was made possible by the generous support of the Global Disaster Preparedness Center and Response 2 Resilience. The funders had no role in study design, data collection and analysis, decision to publish, or preparation of the manuscript. The views presented in this paper are solely those of the researchers and are completely independent of the funder.

## Data Availability

All relevant data are within this manuscript.

## Corresponding Author

Stephen Apanga

Email: apangastephen@hotmail.com

University for Development Studies, Department of Community Health and Family Medicine School of Medicine and Health Sciences

## APPENDIX

**A demonstration of community scorecard scoring process:**